# Transition of Online Adaptive Stereotactic Radiotherapy for Pancreatic Cancer From Magnetic Resonance-Guided Linear Accelerator (MR-Linac) to State-of-the-Art Cone-Beam Computed Tomography (CBCT)

**DOI:** 10.7759/cureus.68386

**Published:** 2024-09-01

**Authors:** Laura A Rechner, Mette Felter, Susanne Bekke, Susan Biancardo, Marianne F Rønjom, Mette Pedersen, David Sjöström, Inna M Chen, Patrik Sibolt

**Affiliations:** 1 Department of Oncology, Copenhagen University Hospital-Herlev and Gentofte, Herlev, DNK

**Keywords:** stereotactic body radiotherapy (sbrt), stereotactic, pancreatic cancer, adaptive, oart

## Abstract

Pancreatic cancer is one of the most challenging tumor sites to treat safely and effectively with radiotherapy due to the anatomical location and aggressiveness of the disease. One modality that has shown promising results, which our institution has been employing, is online adaptive stereotactic radiotherapy using a magnetic resonance-guided linear accelerator (MR-linac). However, due to unforeseen circumstances regarding our MR-linac, it was necessary for our institution to use an alternative treatment technique. In this case report, we describe our experience using our ring-gantry linac equipped with an advanced cone-beam computed tomography (CBCT) system to treat a 61-year-old patient with advanced pancreatic cancer with CBCT-guided online adaptive stereotactic radiotherapy. In a short time period of four weeks, we prepared for this case by training with the surface scanning motion management system and developing procedures for planning and adaptation. The patient was prescribed 24 Gy in three fractions, with a risk-adapted approach using strict organ-at-risk (OAR) constraints. Daily CBCT was used for online adaptation of the plan, and the superior plan (non-adapted or adapted) was selected for treatment. For this patient, the adapted plan was chosen for all three fractions, due to OAR constraints being violated in the non-adapted plan. In summary, we found that for this patient, high-quality CBCT guidance for daily re-contouring, in combination with motion management, enabled the use of daily adaptive radiotherapy to safely deliver stereotactic radiotherapy. The results from this case report are promising, and CBCT-guided online adaptive stereotactic radiotherapy for pancreatic cancer warrants further investigation in more patients.

## Introduction

Delivering radiotherapy to patients with tumors in the pancreas is challenging due to the aggressive nature of the disease and proximity to critical organs at risk (OARs) ​[1,2]​ . Conventionally fractionated radiotherapy has shown limited benefit ​[3,4]​ and stereotactic body radiotherapy (SBRT) for locally advanced pancreatic cancer has historically shown mixed results with limited benefit ​[5]​ or unacceptable toxicity ​[6,7]​. However, a recent analysis of patients with tumors located more than 1 cm from critical OARs who received higher doses demonstrated more favorable outcomes ​[8]​. This provides the rationale for testing a dose-escalating strategy for tumors close to OARs, by using a risk-adapted approach prioritizing OARs over target coverage and advanced treatment techniques. One technique that has shown promising results for overall survival and toxicity is SBRT with daily magnetic resonance (MR)-guided adaptation ​[9-11]​. 

At our institution, a clinical protocol for advanced pancreatic cancer combining immunotherapy, chemotherapy, and SBRT was ongoing between 2020 and 2023 (Phase 1/2 Study in Borderline Resectable, Locally Advanced or Metastatic Pancreatic Cancer To Assess Safety and Potential Efficacy of Dual Checkpoint Inhibition in Combination with Gemcitabine and Nab-paclitaxel Followed by Immune-Chemoradiation (LAPTOP): NCT04247165). The SBRT for the protocol prescribed a dose of 24 Gy/three fractions, delivered using daily online adaptive radiotherapy with strict OAR constraints. A 0.35T MR-linear accelerator (linac) (ViewRay Inc., Oakwood, USA) was the treatment modality used for delivery of the SBRT, due to the ability to perform online adaptive radiotherapy (oART) and gate the treatment on internal anatomy. However, there was a time period immediately following the bankruptcy of the company ​[12]​ when we were not clinically using the system due to practical and legal concerns.

## Case presentation

Patient presentation 

**Figure 1:**
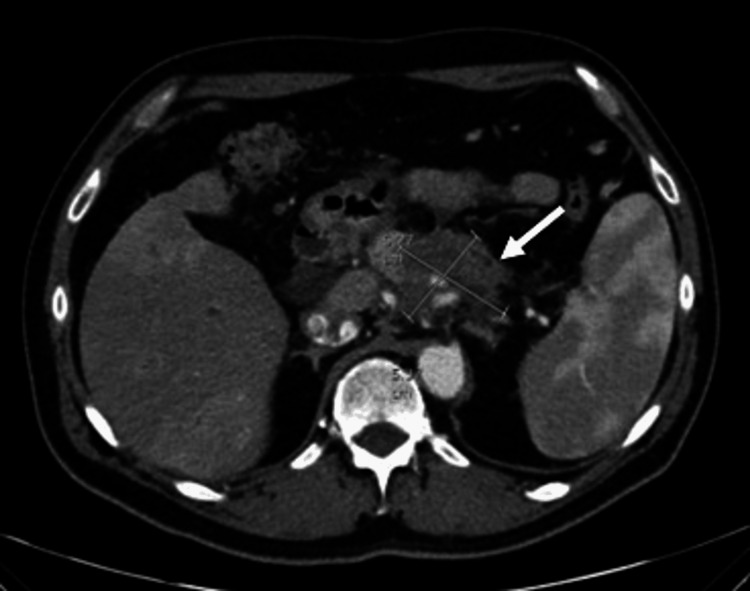
Diagnostic CT showing the patient’s advanced pancreatic tumor CT: Computed tomography

During the time period when the MR-linac at our institution was not in clinical use, the last patient in the LAPTOP protocol was scheduled for SBRT. The patient was a 61-year-old male referred to our department in August 2023 with locally advanced pancreatic cancer. At the referral, he was suffering from weight loss and upper abdominal pain. The tumor was 5 cm in diameter, located in the corpus pancreatic with involvement of the lienal vessels and truncus coeliacus (Figure 1). During staging according to the protocol, liver metastases were identified on CT and confirmed by an MRI of the liver. After two months of the introduction of gemcitabine, nab-paclitaxel, nivolumab, and ipilimumab, the patient was planned for SBRT of the pancreatic tumor concomitant with chemotherapy and nivolumab according to the protocol. 

Choice of treatment technique 

To provide the patient with the best possible care, the combination of high-quality daily image guidance, daily adaptation, and respiratory motion management was required; thus, our adaptive ring-gantry linac (Ethos, Varian, a Siemens Healthineers Company, Palo Alto, CA) was chosen for treatment ​[13]​. The cone-beam computed tomography (CBCT) imaging system in our ring-gantry linac had been recently upgraded (HyperSight, Varian, a Siemens Healthineers Company), providing CBCT with high resolution and image contrast and the ability to acquire the scan within a single breath-hold ​[14]​. Furthermore, a surface scanning system (IDENFITY, Varian, a Siemens Healthineers Company) was available to provide a method for respiratory motion management. However, our institution did not yet have clinical experience with stereotactic treatments or motion management with this system. Fortunately, due to the timing of the multimodality regime of the LAPTOP protocol, there were four weeks between the initial referral of the patient and the planned start date for radiotherapy. Therefore, in preparation for this case, vendor training on the surface scanning system was immediately arranged and information about the treatment technique was gathered from experts in the field ​[15]​. 

Simulation, planning, and preparation for treatment

**Figure 2:**
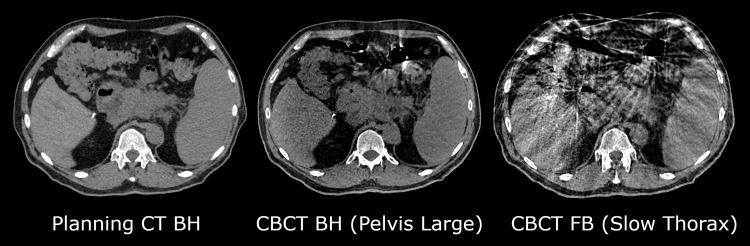
Axial slices at the level of the tumor showing the difference between image quality of the simulation planning CT acquired in BH (left), the HyperSight CBCT acquired in BH (middle, six-second pelvis large protocol), and the CBCT acquired in FB, where artifacts are more pronounced on the FB scan (right, thorax slow protocol) BH: Breath-hold; FB: free breathing; CT: computed tomography; CBCT: cone-beam computed tomography

The patient was immobilized with a vacuum bag with both arms up and was simulated with CT with contrast, MRI, and CBCT. Before CT simulation, the patient performed breath-hold training, where the initial goal was to perform expiration breath-hold, but the patient felt more comfortable performing a mid-inspiration breath-hold. Four repeated breath-hold CT scans (no contrast, arterial phase, late arterial phase, and venous phase) and a 4DCT were acquired. After CT simulation, the patient was also scanned in the treatment position using a 1.5T scanner with T2, diffusion, and T1, and Dixon sequences. While the patient was receiving the MRI simulation scans, the CT scans were imported to the planning system (Ethos v1.1MR1, Varian, a Siemens Healthineers Company), and a dummy plan was created for the treatment system to enable saving the CBCTs acquired during simulation in the clinical system. Additionally, the body surface from a breath-hold CT scan was exported to the surface scanning system, and a region of interest was pre-defined across the upper abdomen/lower thorax region. The threshold for the vertical surface agreement was set to 1-2 mm (corresponding to a gating window of 2-4 mm). Four repeated breath-hold CBCTs were acquired on the treatment unit (six-second pelvis large CBCT protocol, 140 kV) and one free breathing CBCT (thorax slow protocol, 120 kV) was acquired (Figure 2). The repeatability of the breath-hold CTs and CBCTs was reviewed,and the uncertainty in tumor position was assessed to be under 5 mm, which is similar to what is reported in the literature ​[16]​. 

**Figure 3:**
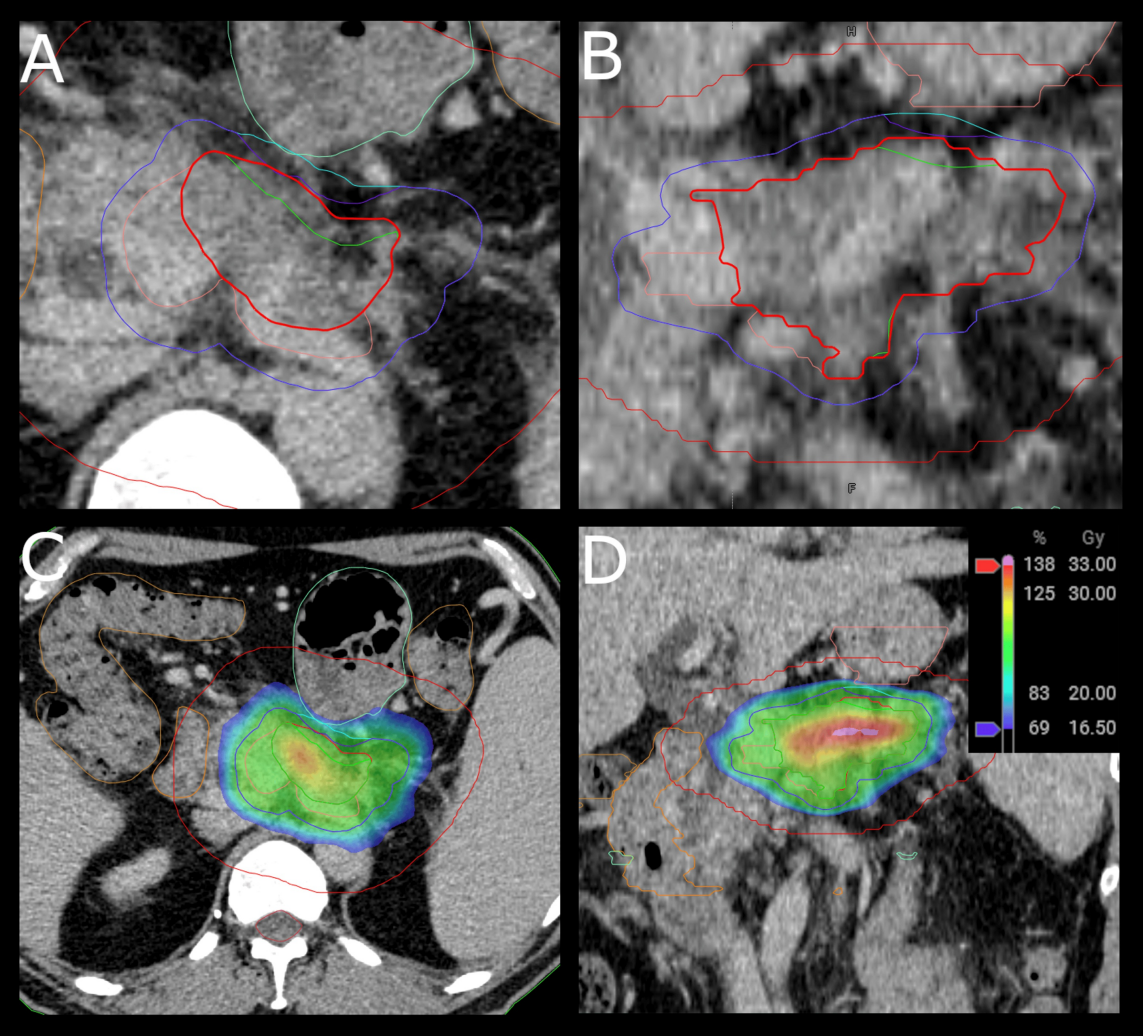
Axial and coronal views of the contours (axial: panel A; coronal: B) for the reference treatment plan, using a dose colorwash scale from 16.5 to 33 Gy (axial: panel C; coronal: panel D) GTV: Gross tumor volume; CTV: clinical target volume (CTV); PTV: planning target volume; opt: optimization Structures are shown as follows: GTV in red, GTV_opt in bright green, CTV in pink, PTV in cyan, PTV_opt in dark blue, contour ring in red, large bowel in tan, small bowel in light green, duodenum in orange, stomach in pink, spinal cord in red, body in green. Differences in GTV and GTV_opt and PTV and PTV_opt, respectively, can be seen anteriorly (panel A) and cranially (panel B), where coverage is compromised to protect the small bowel (panel C) and stomach (panel D)

A treatment plan was created for a prescription dose of 24 Gy in three fractions (Figure 3). A volumetric modulated arc therapy (VMAT) plan with two three-quarter arcs (from 270° to 179°) was created in another treatment planning system to set the geometry (Eclipse v16.1, Varian, a Siemens Healthineers Company) and then imported into the adaptive treatment planning system (Ethos) and reoptimized using a planning template. A uniform expansion of 5 mm was used from the clinical target volume (CTV) to the planning target volume (PTV). Treatment planning used a risk-adapted approach ​[15]​, where optimization structures were used, and target coverage was sacrificed in order to achieve hard constraints for OARs (Table 1). A 10 mm margin was used from critical OARs near the target (duodenum, stomach, small bowel, and large bowel) to the optimization gross tumor volume (GTV_opt), and a 5 mm margin was used for the optimization planning target volume (PTV_opt) ​[17]​. Additional OARs considered during treatment planning were the kidneys, spinal cord, and skin. A normalization method to meet all highest-priority OAR constraints was used to ensure OAR constraints were always achieved. A contour ring was created as an expansion of the PTV (3 cm radially, 1.5 cm craniocaudally) to focus recontouring during daily adaptation to the immediate region surrounding the tumor. To be able to propagate the contour ring rigidly and see the ring during contouring, it was created as a target in the planning system. 

**Table 1:** Dose-volume constraints and metrics for critical organs at risk, comparing the original plan with the scheduled plan (non-adapted) and adapted plan for each fraction Constraints that were not achieved are shown in bold. All constraints were achieved for the adapted plans but not the scheduled plans. The plan chosen for treatment is shown with an asterisk (*), which was the adapted plan for all fractions

Organ	Constraint	Original plan (cm^3^)	Fraction 1		Fraction 2		Fraction 3	
			Scheduled (cm^3^)	Adapted* (cm^3^)	Scheduled (cm^3^)	Adapted* (cm^3^)	Scheduled (cm^3^)	Adapted* (cm^3^)
Small bowel	V25.2 Gy <= 0.5cm3	0.0	0.22	0.0	0.5	0.0	0.02	0.0
	V17.7 Gy <= 5.0cm3	0.33	11.48	0.42	9.16	0.26	4.50	0.53
Duodenum	V22.2 Gy <= 0.5cm3	0.0	0.0	0.0	0.0	0.0	0.58	0.0
	V16.5 Gy <= 5.0cm3	0.02	0.0	0.0	0.46	0.12	4.04	1.11
Stomach	V22.2 Gy <= 0.5cm3	0.0	0.0	0.0	0.18	0.0	2.62	0.0
	V16.5 Gy <= 10.0cm3	0.18	0.0	0.0	5.20	1.94	8.27	2.19
Large bowel	V28.2 Gy <= 0.5cm3	0.0	0.0	0.0	0.0	0.0	0.0	0.0

The plan and simulation CBCTs were exported to a treatment emulator system to test adaptation before the patient’s first scheduled fraction. In this test, the scheduled plan did not achieve the constraint for the stomach (1.68 cm^3 ^receiving 22.2 Gy, constraint of 0.5 cm^3^), but the reoptimization for the adapted plan was able to achieve this constraint (0.0 cm^3^). The treating physicians were also able to both practice using the tools (as their previous experience was with the MR-linac treatment system) and assess that the image quality of the simulation CBCTs was high enough to confidently perform online contouring and adaptation. 

Online adaptive process and dosimetric results 

**Figure 4:**
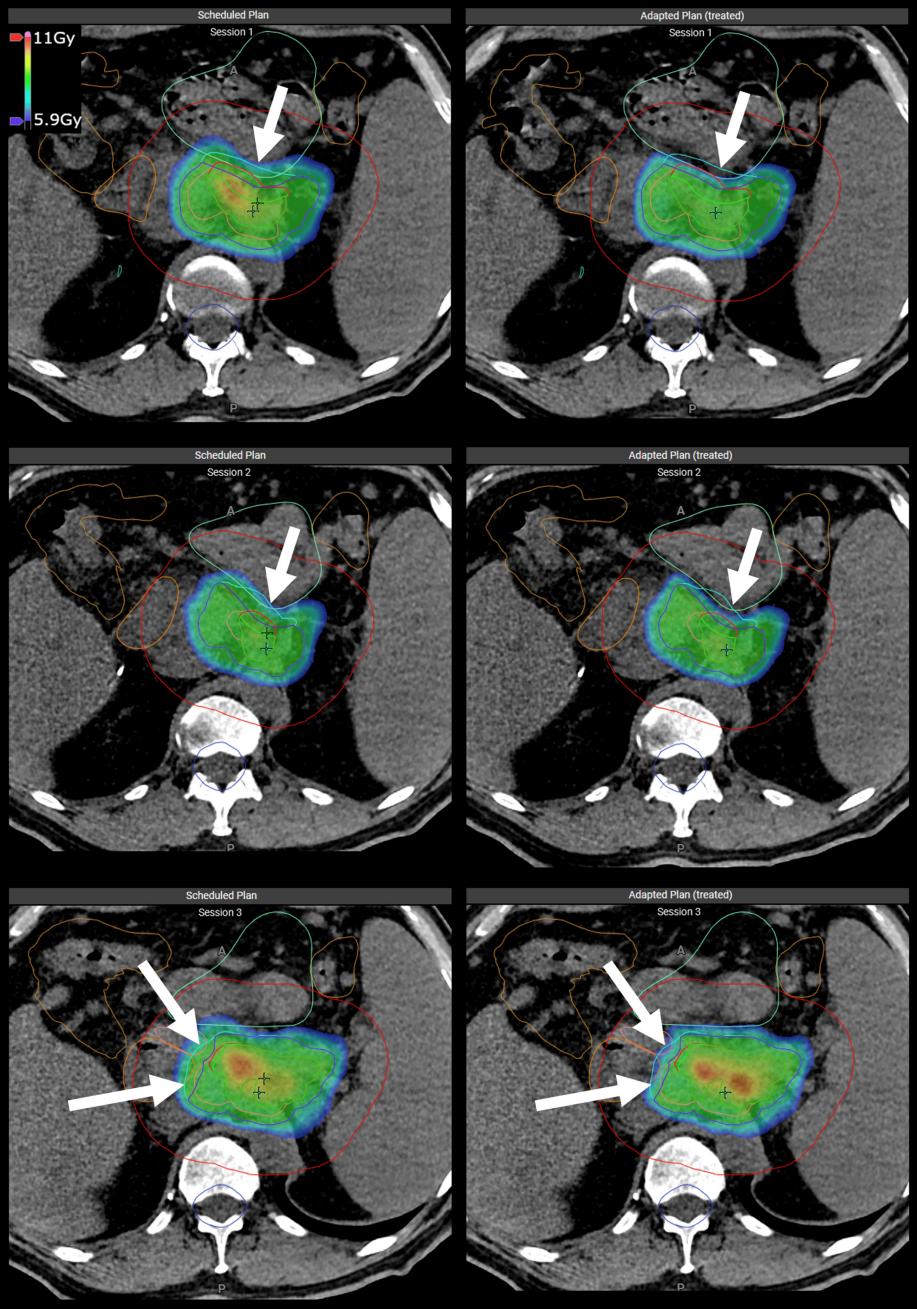
Axial slices showing a comparison of the scheduled (non-adapted) plans (left) versus the adapted plans (right) for fractions 1 (top), 2 (middle), and 3 (bottom). Arrows show the regions where the adapted plan reduced the dose to OARs. The colorwash scale from 5.9 to 11 Gy (fraction dose) OARs: Organs at risk; GTV: gross tumor volume; CTV: clinical target volume; PTV: planning target volume; opt: optimization; PRV: planning risk volume Structures are shown as follows: GTV in red, GTV_opt in bright green, CTV in pink, PTV in cyan, PTV_opt in dark blue, contour ring in red, large bowel in tan, small bowel in light green, duodenum in orange, stomach in pink, spinal cord PRV in blue, body in green

The patient was positioned using lasers and then practiced breath-hold using visual feedback from the surface scanning system. A threshold of 1 mm was used for the surface for treatment (2 mm gating window). A CBCT was acquired during a single breath-hold, and the surface reset during the same breath-hold so that the surface used for treatment corresponded to the exact surface during imaging. After reviewing the quality of the CBCT, the contour ring and target were rigidly propagated from the planning CT to the CBCT and matched. Next, contours were edited to fit the daily anatomy, with special focus on OARs inside the contour ring near the target. After the reoptimization, the scheduled (non-adapted) and adapted plans were reviewed, and the superior plan was chosen for treatment (Table 1). For all fractions, the adapted plan was chosen (Figure 4). 

To verify the positioning after the elapsed time for the adaptive process, a verification CBCT was acquired before treatment. If the difference in tumor position was ≥3mm, a shift was applied during breath-hold, new surface acquired after the shift during the same breath-hold, and another verification CBCT was acquired. If the difference was <3mm, no shift was applied, and treatment was continued. The patient was asked to perform breath-hold, and the beam was manually gated to deliver dose only when the patient was within the gating window for a maximum of 20 seconds. Before the second arc, a verification CBCT was acquired midway, with the same procedure for shifts. Shifts were only required to be made once: during the midway CBCT for fraction 2. Delivery time for each arc was approximately 10 minutes, and the overall treatment time was approximately an hour and 20 minutes (Table 2), which is within the 90-minute timeslot that was standard for MR-linac treatments.

**Table 2:** Timepoints during treatment. The times listed for the first CBCT are relative to the beginning of the treatment session and thereafter relative to each timepoint. The total elapsed time is the sum of the elapsed times CBCT: Cone-beam computed tomography

Timepoints	Fraction 1 (hr:min:sec)	Fraction 2 (hr:min:sec)	Fraction 3 (hr:min:sec)
Patient alignment, IDENFITY preparation, first CBCT	00:22:43	00:24:29	00:20:08
Structure propagation and editing, reoptimization, plan selection	00:26:18	00:28:51	00:37:51
Pretreatment CBCT, treatment of first arc, first midway CBCT	00:11:47	00:14:58	00:13:02
Realignment if necessary, treatment of second arc, end of treatment	00:13:00	00:13:43	00:10:26
Total elapsed time	01:13:48	01:22:01	01:21:27

Retrospective plan comparison

**Figure 5:**
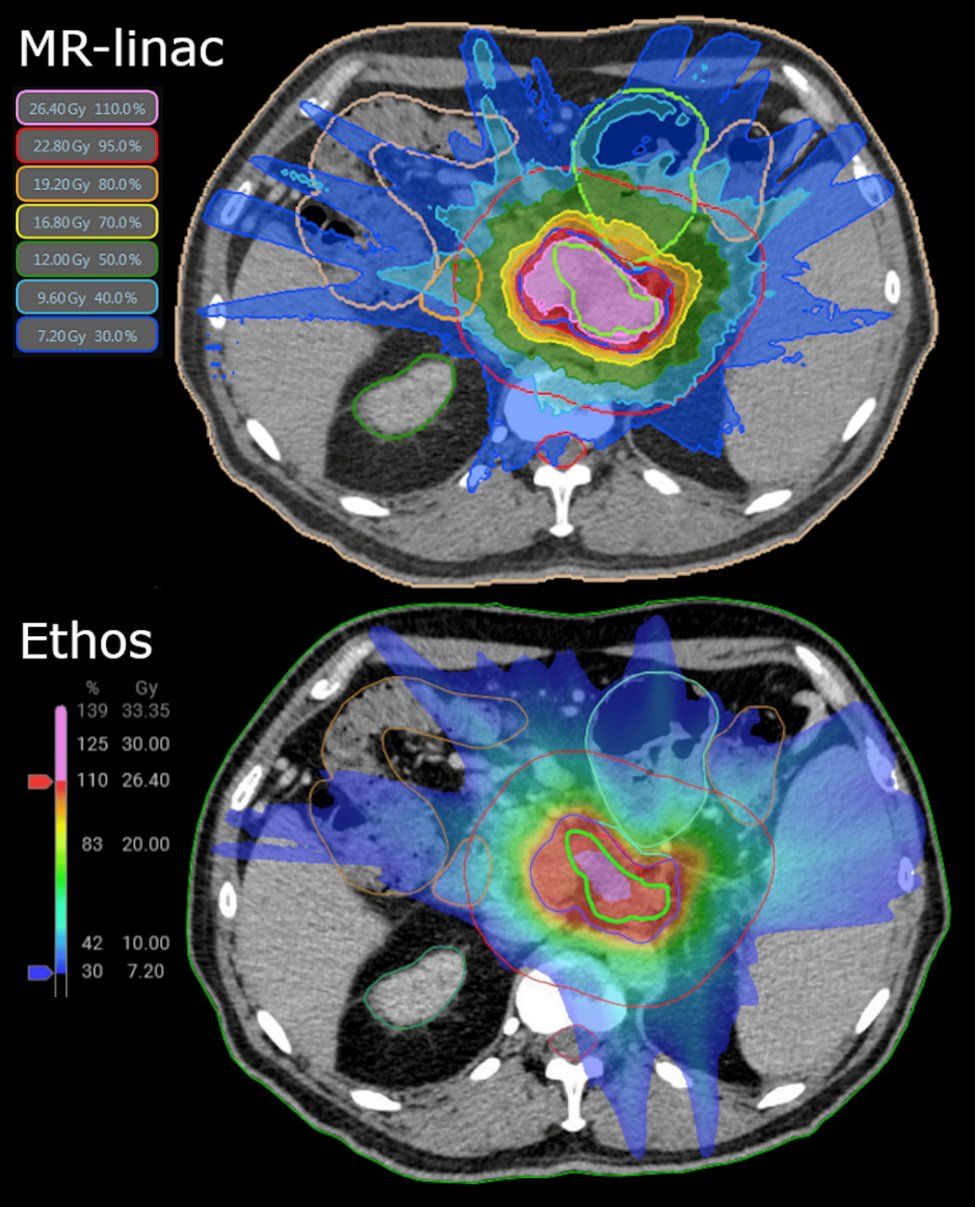
Comparison of dose distribution from an MR-linac plan (top) compared to an Ethos plan (bottom) for this patient case. The colorwash levels are from 30% to 110% of the prescription MR-linac: Magnetic resonance-guided linear accelerator; CTV: clinical target volume; PTV: planning target volume; GTV_opt: optimization gross tumor volume; PTV_opt: optimization planning target volume Structures are shown as follows: GTV in red, GTV_opt in bright green, CTV in pink, PTV in cyan, PTV_opt in dark blue, contour ring in red, large bowel in tan, small bowel in light green, duodenum in orange, stomach in pink, spinal cord in red, body in green/tan

To investigate how the differences in the planning systems impacted the plan quality, a comparison plan was retrospectively created with the MR-linac planning system (ViewRay Inc.) (Figure 5). For the step-and-shoot intensity-modulated radiation therapy (IMRT) MR-linac plan, 25 beams and 54 segments were used. The same 5 mm PTV margin was used for both techniques, but due to differences in how each system performed margin expansions, the resulting volumes were slightly different. For the MR-linac plan, the GTV_opt and PTV_opt were created using a 4.5 mm margin for cropping duodenum and stomach and 3 mm margin for cropping the small bowel and large bowel. This smaller margin resulted in slightly higher percentages of the GTV and PTV being covered by the optimization structures (Table 3). In addition, the possibility to normalize the plan to the limit of an OAR constraint (iso-toxic normalization) for the MR-linac plan (in this case, the small bowel) allowed the plan to be normalized slightly higher than the Ethos plan, yielding some small improvements in coverage of the GTV. However, in contrast, the PTV coverage was slightly better on the Ethos plan. Overall, while there were some small differences in planning technique, optimization, and normalization, the plans were similar, and no large differences in plan quality were observed. 

**Table 3:** Comparison of dose and volume metrics between a plan created with the MR-linac system and the clinically used reference plan made with the Ethos system MR-linac: Magnetic resonance-guided linear accelerator; GTV: gross tumor volume; PTV: planning target volume; opt: optimization

Structure	Metric	MR-linac plan	Ethos plan
GTV	D100%	24.0 Gy	23.3 Gy
	D98%	26.2 Gy	25.3 Gy
	Mean dose	28.1 Gy	27.9 Gy
	Volume	59.8 cm^3 ^	56.9 cm^3 ^
GTV_opt	Volume	59.8 cm^3 ^(100% of GTV)	53.0 cm^3^ (93% of GTV)
PTV	D95%	23.7 Gy	24.5 Gy
	D0.03 cm^3^	31.1 Gy	33.3 Gy
	Volume	139.4 cm^3^	134.9 cm^3^
PTV_opt	Volume	136.6 cm3 (98% of PTV)	131.0 cm^3^ (97% of PTV)
Small bowel	V25.2 Gy	0.0 cm3	0.0 cm^3 ^
	V17.7 Gy	5.0 cm3	0.33 cm^3 ^
Duodenum	V22.2 Gy	0.0 cm3	0.0 cm^3 ^
	V16.5 Gy	1.2 cm3	0.02 cm^3 ^
Stomach	V22.2 Gy	0.0 cm3	0.0 cm^3 ^
	V16.5 Gy	0.0 cm3	0.2 cm^3 ^
Large bowel	V28.2 Gy	0.0 cm3	0.0 cm^3^
Body ring	D1cm^3 ^	14.1 Gy	12.4 Gy

## Discussion

This case report summarizes our experience of converting a highly specialized stereotactic treatment of advanced pancreatic cancer from the MR-linac to CBCT-based oART. One previous case of stereotactic treatment for pancreatic cancer using CBCT-based oART has been reported ​[15]​; however, ours was the first reported case to use a new type of state-of-the-art CBCT (HyperSight, Varian, a Siemens Healthineers Company) for daily imaging. For their case, they also found that the technique was feasible and concluded that clinical trials are warranted to further investigate the modality. 

There are some differences between the procedure using an MR-linac compared to CBCT-based oART. One major difference is the use of 0.35T MRI compared to CBCT for the daily imaging used for contouring and planning. For this case, the physicians tested the ability to contour using the emulator and were satisfied with the quality of the CBCT images, especially with the reduced artifacts using a single-breath-hold image (Figure 2). Another major difference is the method used for motion management. For the MR-linac, the tumor (or a surrogate internal structure) is contoured and tracked during treatment using cine MR imaging, and the beam is gated using a margin around the tracking structure (3 mm as standard practice at our institution). For the CBCT-based workflow using surface scanning, the external surface is assumed to be correlated with the internal anatomy and verified before and midway through treatment. These differences in motion management led us to use slightly different margins for cropping optimization structures for the two techniques: MR-linac plans were typically planned with 4.5-6 mm margins from the stomach and duodenum and 3 mm margins from large and small bowel structures to both the PTV_opt and CTV/GTV_opt, which is similar to what was used in the SMART trial [10]. For the CBCT-based procedure, we used a slightly more conservative approach for those organs with 5 mm margins for PTV_opt and 10 mm margins for GTV_opt. The rationale was to keep the highest dose confined to an area farther from the critical OARs due to the lack of internal tracking. Instead of internal tracking, midway CBCTs were used to verify the intrafraction position of the tumor, where we applied a shift if the difference in position was greater than 3 mm, which was only required during one fraction. These differences in techniques for margins, planning, optimization, and normalization resulted in some slight differences in the treatment plans (Table 3). 

Another difference between the systems involves the procedure for obtaining electron densities for the dose calculation for adaptation. Both the MR-linac system (ViewRay) and the Ethos system (v1.1MR1) start with a deformation of the CT simulation scan to the daily anatomy to convert Hounsfield units to electron densities. In the MR-linac system, the electron density map can be reviewed and edited to perform overrides for inaccuracies, for example, air cavities that are not in the correct location compared to the daily MR scan. In contrast, the Ethos system lacks tools to evaluate or edit the deformed CT (synthetic CT) during the adaptive process, and it is only visible via the quality assurance system Mobius (Varian, a Siemens Healthineers Company, Palo Alto, CA). However, the next version of Ethos (v2.0), which we have now clinically implemented at our institution, uses the daily CBCT (HyperSight) for dose calculation, so the electron densities match the daily anatomy without need for manual corrections. The improved image quality of HyperSight and the use of direct dose calculation on the CBCT further emphasize the potential of using Ethos for challenging adaptive treatments where large changes in densities are possible, for example, air cavity changes in the abdomen and pelvis or tissue density changes in the lung. 

## Conclusions

In this case report, we summarized our experience with converting a treatment previously performed with the MR-linac to a CBCT-based workflow. We report that, for this patient, the treatment was feasible, and the combination of motion management, state-of-the-art CBCT, and daily adaptation enabled the delivery of SBRT in the upper abdomen while adhering to strict OAR constraints. The adapted plan was selected for treatment for all fractions due to OAR constraints, which would have been violated with the non-adapted plan. The promising results from this case study support further investigation of this technique in more patients through clinical trials. 
